# Recurrent Nasal-Type Extranodal Natural Killer/T-Cell Lymphoma with Meningeal Involvement

**DOI:** 10.1155/2021/9972694

**Published:** 2021-07-24

**Authors:** Sylvester Homsy, Ralph Kamel, Mark Raden, Yevgeniy Skaradinskiy

**Affiliations:** ^1^Department of Internal Medicine, Staten Island University Hospital-Northwell Health, Staten Island, New York, NY, USA; ^2^Department of Radiology, Staten Island University Hospital-Northwell Health, Staten Island, New York, NY, USA; ^3^Division of Hematology/Oncology, Staten Island University Hospital-Northwell Health, Staten Island, New York, NY, USA

## Abstract

Extranodal natural killer/T-cell lymphoma nasal type (NNKTL) is a type of non-Hodgkin's lymphoma that has been associated with Epstein–Barr virus (EBV). It has an aggressive behavior, known for predilection to metastasize to different organs. Central nervous system (CNS) spread from a primary location has been reported. Different modalities of treatment such as chemotherapy and radiation therapy have been employed in the management of this disease. Severe toxicities of currently available treatment have made clinicians seek more targeted therapies using molecular profiling. We present a 44-year-old Hispanic patient who was diagnosed with an early-stage NNKTL and treated with the modified SMILE regimen for 6 cycles. His EBV DNA PCR turned undetectable and remained so throughout the treatment. He sustained complete right vision loss due to right optic nerve invasion by the tumor, leading to prophylactic intravitreal methotrexate to the contralateral eye. The patient achieved good response with minimal residual disease. He was supposed to start radiation as a sequential therapy. However, the acute development of severe headache and confusion lead to a complete workup showing leptomeningeal spread. He eventually succumbed to the disease.

## 1. Introduction

Extranodal natural killer/T-cell lymphoma nasal type (NNKTL) occurs primarily in the nasal cavity or paranasal sinuses. It is characterized by ulceration and necrosis of the nasal cavity and midline facial tissues [1]. It is commonly observed in Asia and South America where it accounts for up to 10% of all non-Hodgkin lymphoma. In the United States, it only accounts for 3.3% of all T-cell lymphomas [2]. It is known to have an aggressive course which is often fatal due to rapid local progression and distant metastases mainly to the lymph nodes, lungs, liver, and the gastrointestinal tract [3].Central nervous system (CNS) involvement is rare and has only been reported in 7% of a Japanese cohort at presentation [4]. Herein, we report a case of an otherwise healthy male who was successfully treated for a primary NNKTL in his nasal cavity. He subsequently developed meningeal recurrence that was fatal.

## 2. Case Report

**Figure 1:**
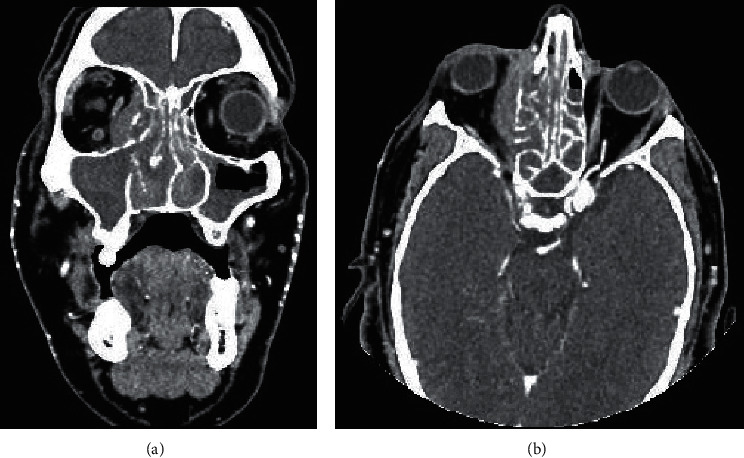
CT maxillofacial with IV contrast showing opacified ethmoid sinuses with bone destruction of the right lamina papyracea with soft-tissue extension into the right orbit. (a) Coronal view; (b) axial view.

**Figure 2:**
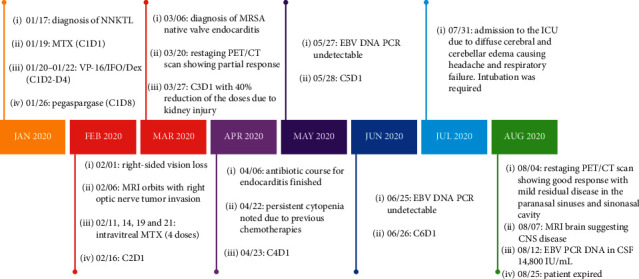
Timeline highlighting diagnosis, treatment schedule, disease course, and findings.

**Figure 3:**
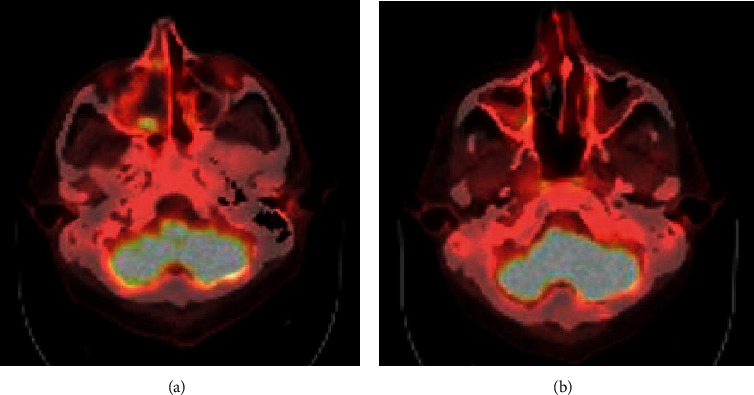
PET/CT scan highlighting the maxillofacial structures. (a) After 2 cycles of mSMILE, a single 1.1 cm focus of increased FDG uptake was seen along the right sinonasal cavity, SUV max 9.3. (b) Restaging PET/CT scan after 6 cycles of mSMILE showing mild residual uptake in the right maxillary sinus, SUV max 4.2.

**Figure 4:**
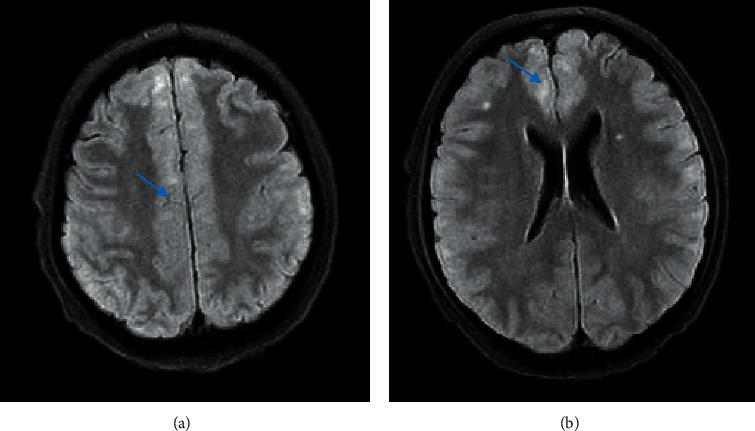
MRI brain with IV contrast showing several scattered bilateral foci of increased T2/FLAIR signal in the bifrontal regions. Also noted, as shown by the blue arrows, was a diffuse swelling of the gyri involving bilateral frontal and anterior parietal lobes.

**Figure 5:**
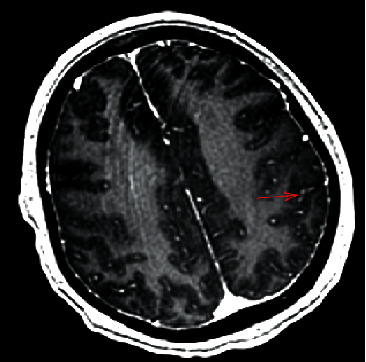
MRI brain with IV contrast suggesting sulcal enhancement of the gyrus representing underlying leptomeningeal disease.

A 44-year-old Hispanic male from Mexico presented to the emergency department for progressive nasal congestion, rhinorrhea, ear fullness, facial swelling, and sore throat that has been ongoing for 2 months. He also noted gradual vision loss of his right eye and intermittent bloody discharge from both the nostrils. Bedsides, rhinoscopy showed swelling of the right nare and nasal turbinates along with small blood clots without active bleeding. A maxillofacial computed tomography (CT) scan showed phlegmonous change about the right lamina papyracea with an erosive change. Also shown was destruction and soft-tissue swelling that extends along the medial border of the right orbit (Figure 1). Biopsy of the right nasal vestibule was diagnostic for extranodal NK/T-cell lymphoma nasal type. The atypical cells were positive for CD2, CD3, CD4, CD56, CD43, and Bcl-2 and negative for CD5, CD7, CD8, CD10, CD20, CD30, and BCL-6. In situ hybridization was positive for the Epstein–Barr encoding region (EBER). Bone marrow biopsy showed a normocellular bone marrow with trilineage hematopoiesis. Additional imaging with magnetic resonance imaging (MRI) of the head showed a vague enhancement over the frontal convexities. EBV DNA PCR in the blood was 4.90 log10 IU/mL (equivalent to 79,792 IU/mL). He was started on a modified SMILE (m-SMILE) regimen consistent of methotrexate 2 g/m^2^ given on day 1, etoposide 100 mg/m^2^, ifosfamide 1500 mg/m^2^, dexamethasone 40 mg/d given on days 2 to 4, and pegasparagase 2000 U/m^2^ given on day 8 of a 28-day cycle (Figure 2). He developed acute right-sided complete vision loss; magnetic resonance imaging of the orbits showed increased enhancement of the optic nerve sheath with a complex edema suspicious for optic nerve invasion. The case was discussed at an ENT oncology and other multidisciplinary oncology conferences; the decision was made to proceed with the same chemotherapy regimen. The patient then received prophylactic intravitreal methotrexate of the left eye. He completed his second cycle of m-SMILE, and a follow-up positron emission tomography-computed tomography (PET/CT) scan showed good response (Figure 3(a)). Methotrexate dose had to be reduced 50% on the third cycle of m-SMILE due to kidney injury. He subsequently received 4 more cycles of m-SMILE with 25% dose reduction of chemotherapeutic agents due to prolonged cytopenia from previous cycles. His EBV DNA PCR turned undetectable after the first cycle and remained so after the final (6^th^) cycle. A restaging PET/CT scan showed good response with faint residual disease in the paranasal sinuses and sinonasal cavity (Figure 3(b)). One month after he completed his last cycle, he developed new-onset severe headache, confusion, and shortness of breath. He was admitted to the emergency department where a CT scan of the head showed diffuse cerebral and cerebella edema. MRI of the brain with intravenous contrast revealed multiple focal cortical and subcortical signal abnormalities, with extensive gyral swelling involving the frontal, anterior parietal, and bilateral temporal lobe. Subtle enhancement in the sulci of the high frontoparietal region, particularly at the central sulcus, was noted (Figures 4 and 5 ). Cerebrospinal fluid examination was positive for CD 56+ cells and aberrant atypical lymphocytes. EBV DNA PCR was 14,800 IU/mL. His course was later complicated by respiratory failure and cerebellar tonsillar herniation; he subsequently succumbed after 19 days of hospitalization.

## 3. Discussion

NNKTL is an EBV-associated malignancy, and it was first reported in 1990 by Harabuchi et al. when EBV DNA PCR was found on the biopsies of nasal T-cell lymphoma of a series of patients diagnosed with this disease [5]. That led to the use of this simple blood test as a marker for tumor progression and surveillance for recurrences [6]. This disease was initially described as “lethal midline granuloma” in the USA because of its destructive property to the nose and face, with progression to a necrotic granuloma [7]. Later on, it has been shown that the neoplasm contains at least 2 lineages of cells: gamma delta T cell and NK cell. This has led to a change in its nomenclature [8]. Initially, diagnosis was made by finding angioinvasion and angiodestruction along with necrosis and apoptosis. However, with the advent of immunophenotyping, a more precise way to diagnose the lymphoma had been since employed [9]. This tumor usually expresses CD2 and CD56 and lacks CD3 (but expresses cytoplasmic CD3*ε*). It is usually EBV positive [10].

One of the most common presentations is nasal obstruction followed by bloody rhinorrhea. B symptoms, including prolonged fevers and weight loss, can also be present in up to 50% of the cases [3, 11]. Ocular involvement represents mainly uveitis/vitritis; orbital infiltration has been reported in the literature as complication of NNKTL [12].CNS involvement has been reported as being a primary involvement as well as a progression or invasion from an existing primary tumor, which is mainly in the nasopharynx. In both instances, the prognosis is dismal and the overall survival drops from 20 months to 7 months [13]. Treatment of NNKTL depends on the stage of the disease at diagnosis. It usually involves chemotherapy, radiation, and bone marrow transplantation [14]. In this case, the patient had limited-stage disease (IE).This case was presented in the tumor board and ENT oncology sessions. The review of the questionable slight enhancement on the initial staging MRI of the head was deemed not significant and unlikely representing CNS disease. The plan was sequential therapy with chemotherapy (modified SMILE regimen) followed by radiation therapy, the so called “sandwich” approach. The decision was to proceed with regular-dose methotrexate (2 g/m^2^). The higher dose of 3 g/m^2^ has a better CNS penetration [15]. Radiation was supposed to be given at a total dose of 50 Gy to the involved paranasal sinuses via intensity-modulated radiation therapy (IMRT) over 5 to 6 weeks. Compliance to clinical appointments as well as chemotherapy induced adverse events such as prolonged neutropenia and fatigue delayed the process. A restaging PET/CT scan performed after the 6^th^ cycle did not show any evidence of disease. The last hospital admission was shortly after the restaging PET scan was performed. It was until the patient was readmitted for headache and confusion that the MRI head was performed and showed evidence of leptomeningeal spread, which was late in the course. To note, that patient had complete right vision loss due to right optic nerve invasion by the tumor and received prophylactic intravitreal methotrexate in the left eye. To date, there is no consensus regarding treating ocular involvement as a CNS involvement with high-dose methotrexate, intrathecal methotrexate, or brain radiation.

## 4. Conclusions

NNKTL is an aggressive type of lymphoma that can spread to the CNS even when diagnosed at an earlier stage. A high index of suspicion is required when faced with a new onset of neurological symptoms where a more aggressive approach such as intravitreal injection of methotrexate and higher doses of its intravenous administration should be used. Although intensive chemotherapy and radiation therapy have provided good outcomes when it comes to survival, their toxicities remain the major limiting factor in the course of treatment; dose and schedule modifications should be proposed without affecting its benefits.

## Additional Points

Sylvester Homsy is the author guarantor.

## References

[1] Harabuchi Y., Takahara M., Kishibe K., Moriai S., Nagato T., Ishii H. (2009). Nasal natural killer (NK)/T-cell lymphoma: clinical, histological, virological, and genetic features. *International Journal of Clinical Oncology*.

[2] Adams S. V., Newcomb P. A., Shustov A. R. (2016). Racial patterns of peripheral T-cell lymphoma incidence and survival in the United States. *Journal of Clinical Oncology*.

[3] Harabuchi Y., Imai S., Wakashima J. (1996). Nasal T‐cell lymphoma causally associated with Epstein‐Barr virus: clinicopathologic, phenotypic, and genotypic studies. *Cancer: Interdisciplinary International Journal of the American Cancer Society*.

[4] Oshimi K., Kawa K., Nakamura S. (2005). NK-cell neoplasms in Japan. *Hematology*.

[5] Harabuchi Y., Yamanaka N., Kataura A., Imai S., Kinoshita T., Osato T. (1990). Epstein-Barr virus in nasal T-cell lymphomas in patients with lethal midline granuloma. *Lancet*.

[6] Ishii H., Ogino T., Berger C. (2007). Clinical usefulness of serum EBV DNA levels of BamHI W and LMP1 for Nasal NK/T-cell lymphoma. *Journal of Medical Virology*.

[7] Harabuchi Y., Kataura A., Kobayashi K. (1992). Lethal midline granuloma (peripheral T‐cell lymphoma) after lymphomatoid papulosis. *Cancer*.

[8] Nagata H., Konno A., Kimura N. (2001). Characterization of novel natural killer (NK)-cell and gamma delta T-cell lines established from primary lesions of nasal T/NK-cell lymphomas associated with the Epstein-Barr virus. *Blood*.

[9] Greer J. P., Kinney M. C., Loughran T. P. (2001). T cell and NK cell lymphoproliferative disorders. *Hematology (ASH Education Program)*.

[10] Jaffe E. S., Chan J. K., Su I. J. (1996). Report of the workshop on nasal and related extranodal angiocentric T/natural killer cell lymphomas. Definitions, differential diagnosis, and epidemiology. *American Journal of Surgical Pathology*.

[11] Wu X., Li P., Zhao J. (2008). A clinical study of 115 patients with extranodal natural killer/T-cell lymphoma, nasal type. *Clinical Oncology*.

[12] Hon C., Kwok A. K., Shek T. W., Chim J. C., Au W. Y. (2002). Vision-threatening complications of nasal T/NK lymphoma. *American Journal of Ophthalmology*.

[13] Kommalapati A., Tella S. H., Ganti A. K., O Armitage J. (2018). Natural killer/T-cell neoplasms: analysis of incidence, patient characteristics, and survival outcomes in the United States. *Clinical Lymphoma Myeloma and Leukemia*.

[14] Allen P. B., Jo Lechowicz. M. (2019). Management of NK/T-cell lymphoma, nasal type. *Journal of Oncology Practice*.

[15] Reni M., Ferreri A. J., Guha-Thakurta N. (2001). Clinical relevance of consolidation radiotherapy and other main therapeutic issues in primary central nervous system lymphomas treated with upfront high-dose methotrexate. *International Journal of Radiation Oncology^∗^Biology^∗^Physics*.

